# Dataset for the quantitative proteomics analysis of the primary hepatocellular carcinoma with single and multiple lesions

**DOI:** 10.1016/j.dib.2015.08.036

**Published:** 2015-09-08

**Authors:** Xiaohua Xing, Yao Huang, Sen Wang, Minhui Chi, Yongyi Zeng, Lihong Chen, Ling Li, Jinhua Zeng, Minjie Lin, Xiao Han, Jingfeng Liu, Xiaolong Liu

**Affiliations:** a The Liver Center of Fujian Province, Fujian Medical University, Fuzhou 350025, People’s Republic of China; b The United Innovation of Mengchao Hepatobiliary Technology Key Laboratory of Fujian Province, Mengchao Hepatobiliary Hospital of Fujian Medical University, Fuzhou 350025, People’s Republic of China; c Liver Disease Center, The First Affiliated Hospital of Fujian Medical University, Fuzhou 350007, People’s Republic of China; d Biotechnology Research Institute, Chinese Academy of Agricultural Sciences, Beijing 100081, People’s Republic of China

## Abstract

Hepatocellular Carcinoma (HCC) is one of the most common malignant tumor, which is causing the second leading cancer-related death worldwide. The tumor tissues and the adjacent noncancerous tissues obtained from HCC patients with single and multiple lesions were quantified using iTRAQ. A total of 5513 proteins (FDR of 1%) were identified which correspond to roughly 27% of the total liver proteome. And 107 and 330 proteins were dysregulated in HCC tissue with multiple lesions (MC group) and HCC tissue with a single lesion (SC group), compared with their noncancerous tissue (MN and SN group) respectively. Bioinformatics analysis (GO, KEGG and IPA) allowed these data to be organized into distinct categories. The data accompanying the manuscript on this approach (Xing et al., J. Proteomics (2015), http://dx.doi.org/10.1016/j.jprot.2015.08.007[1]) have been deposited to the iProX with identifier IPX00037601.

## Specifications table

**Table:** 

Subject area	Biology
More specific subject area	Proteomics on the Hepatocellular Carcinoma
Type of data	List of identified proteins as tables (.xls), raw data in website
How data was acquired	The data was acquired by Liquid chromatography mass spectrometry in tandem (LC–MS/MS).The samples were separated by a Acquity UPLC system (Waters Corporation, Milford, MA) and detected by a Nano-Aquity UPLC system (Waters Corporation, Milford, MA) connected to a quadrupole-Orbitrap mass spectrometer (Q-Exactive) (Thermo Fisher Scientific, Bremen, Germany).
Data format	Filtered and analyzed
Experimental factors	Non-applied
Experimental features	Proteins were extracted from tumor tissues of HCC patients with single and multiple lesions, iTRAQ labeled and then prepared for liquid chromatography-mass spectrometry (LC–MS/MS) analysis.
Data source location	Fuzhou, China, Mengchao Hepatobiliary Hospital of Fujian Medical University
Data accessibility	Filtered and analyzed data are supplied here and raw data have also been deposited to the integrated Proteome resources (iProX) with identifier IPX00037601 (http://www.iprox.org/index).

## Value of the data

• The proteome of hepatocellular carcinoma with single and multiple lesions analyzed using iTRAQ technology.
• A total of 5513 proteins (FDR of 1%) were identified which correspond to roughly 27% of the total liver proteome.
• The in-depth proteomics analysis of the HCC tumor tissues with a single and multiple lesions might be useful for further study of the mechanisms.

## 1 Data, experimental design, materials and methods

### 1.1 Data and experimental design

The data show the lists of proteins identified and quantified in the HCC tumor tissues with single and multiple lesions. The tissues were divided into 4 groups: cancerous tissues from HCC patients with multiple observed lesions (MC group, *n*=30); surrounding noncancerous tissues from HCC patients with multiple observed lesions (MN group, *n*=30); cancerous tissues from primary HCC patients with a single observed lesion (SC group, *n*=30); surrounding noncancerous tissues from primary HCC patients with a single observed lesion (SN group, *n*=30). The detailed characteristics of the selected HCC patients were listed in Table 1. For each group, every 5 individual samples with equal tissue weight were mixed, and then the proteins were extracted from the mixed samples. And then the samples were labeled with the iTRAQ 8-plex reagent as follows: four groups (MC group, MN group, SC group and SN group) were labeled with 113, 114, 115 and 116 isobaric tag, respectively; and the peptides from the biological repetitions of the above 4 groups were labeled with 117, 118, 119 and 121, respectively. The iTRAQ 8-plex labeling was independently repeated 3 times, defining as A, B and C. So we have 6 repeated protein extracts for each group to minimize the individual differences of the patients.

### 1.2 Materials and methods

Tissue samples, including the cancerous and surrounding noncancerous tissues, were obtained from 30 primary HCC patients with multiple observed lesions and 30 primary HCC patients with a single observed lesion, respectively. All patients have undergone radical surgery at Mengchao Hepatobiliary Hospital of Fujian Medical University from August 2010 to January 2013. The protein from these two type HCC tissues was determined by BCA assay (TransGen Biotech, Beijing, China) following the manufacture’s protocol. Afterwards, 100 μg proteins per condition were treated with DTT (8 mM) and iodoacetamide (50 mM) for reduction and alkylation. Afterwards, the proteins were typically digested by sequence-grade modified trypsin (Promega, Madison, WI), and then the resultant peptides mixture was further labeled using chemicals from the iTRAQ reagent kit (AB SCIEX, USA).

The peptide mixture was fractionated by high pH separation using a Acquity UPLC system (Waters Corporation, Milford, MA) connected to a reverse phase column (BEH C18, 1.7 µm, 2.1×50 mm^2^, Waters Corporation, Milford, MA). High pH separation was performed using a linear gradient starting from 5% B to 35% B in 20 min (solution B: 20 mM ammonium formate in 90% ACN, the pH was adjusted to 10.0 with ammonium hydroxide). The column flow rate was maintained at 600 μl/min and column temperature was maintained at room temperature. Finally 40 fractions were collected, and two fractions with the same time interval were pooled together to reduce the fraction numbers, such as 1 and 21, 2 and 22, and so on [2]. Twenty fractions at the end were dried in a vacuum concentrator for further usage.

The fractions were then separated by nano-LC and analyzed by on-line electrospray tandem mass spectrometry. The experiments were performed on a Nano-Aquity UPLC system (Waters Corporation, Milford, MA) connected to a quadrupole-Orbitrap mass spectrometer (Q-Exactive) (Thermo Fisher Scientific, Bremen, Germany) equipped with an online nano-electrospray ion source. 8 μl peptide sample was loaded onto the trap column (Thermo Scientific Acclaim PepMap C18, 100 μm×2 cm)with a flow of 10 μl/min, and subsequently separated on the analytical column (Acclaim PepMap C18, 75 μm×50 cm) with a linear gradient, from 2% D to 40% D in 135 min (solution D: 0.1% formic acid in ACN). The Q-Exactive mass spectrometer was operated in the data-dependent mode to switch automatically between MS and MS/MS acquisition. Survey full-scan MS spectra (*m*/*z* 350–1200) was acquired with a mass resolution of 70 K, followed by 15 sequential high energy collisional dissociation (HCD) MS/MS scans with a resolution of 17.5 K. In all cases, one microscan was recorded using dynamic exclusion of 30 s.

### 1.3 Data analysis

**Fig. 1:**
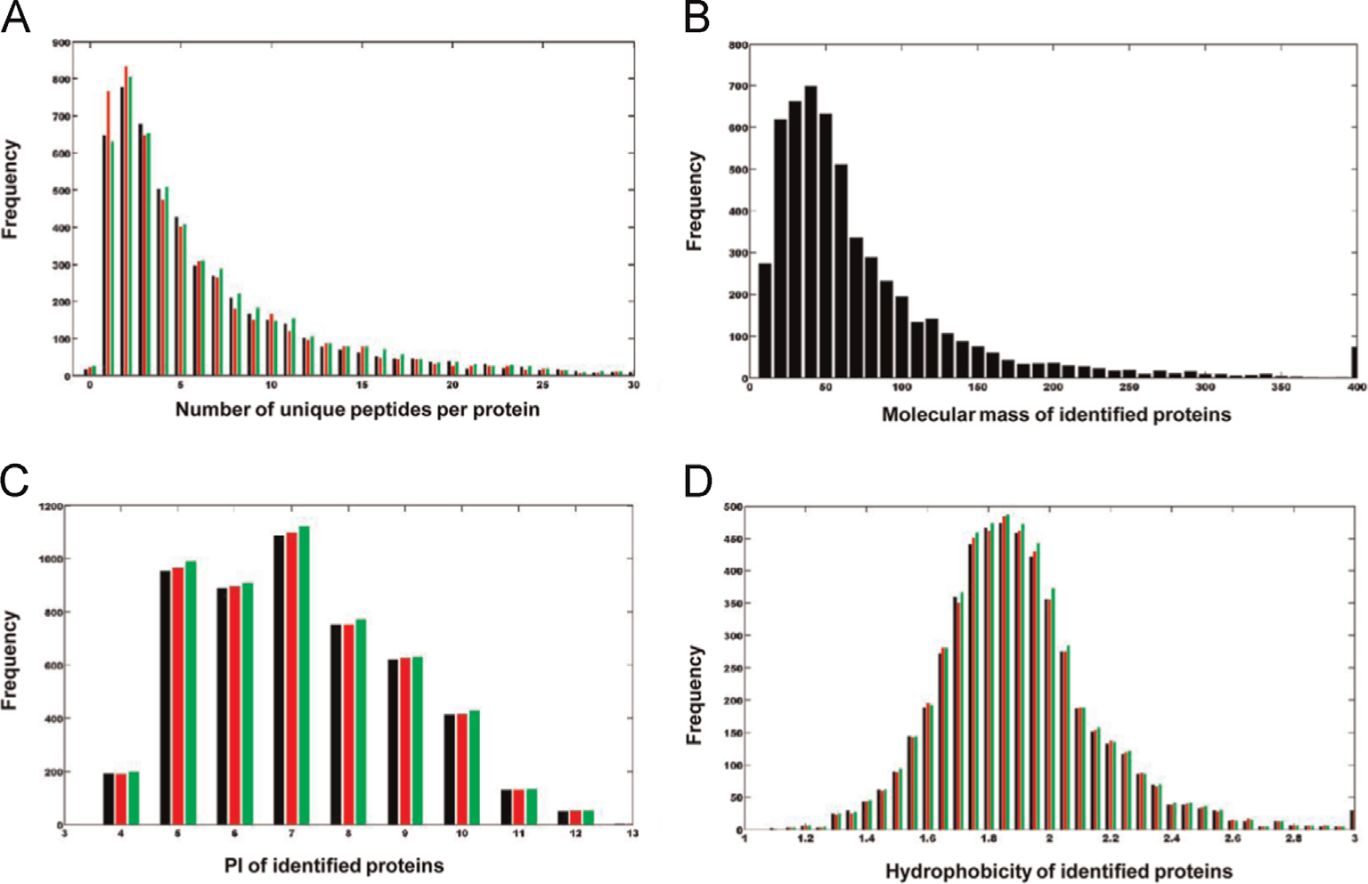
The qualities of the proteome dataset. (A) Frequency distribution of the identified proteins with ≥1 unique peptides. (B) Molecular weight distribution of identified proteins proved that there is no bias in the protein extraction process. (C) Isoelectric point distribution of the identified proteins to show the unbias of the protein extraction. (D) Protein hydrophobicity distribution of the identified proteins.

All the raw files generated by the Q-Exactive instrument were converted into mzXML and MGF files using the ms convert module in Trans-Proteomic Pipeline (TPP 4.6.2). All MGF files were searched using Mascot (Matrix Science, London, UK; version 2.3.0) against a human_database provided by The Universal Protein Resource (http://www.uniprot.org/uniprot, released at 2014-04-10, with 20,264 entries). Using the results from Scaffold_4.3.2, we quantified 5513 proteins in three iTRAQ 8-plex labeling replicates. The complete list of identified proteins in our dataset is shown in Table S1. The detailed characteristics of proteomes of the primary HCC with single and multiple lesions, including Molecular Weight (MW), Isoelectric Point (PI), Hydrophobicity, exponentially modified Protein Abundance Index (emPAI), Quantitative Clustering, Average Coefficient of Variance (CV), quantification results with percentage variability, were included in the list as well. The distribution of unique peptide numbers per protein, MW, PI and hydrophobicity also clearly showed that the overall proteome datasets of the primary HCC with single and multiple lesions had no strong bias (Fig. 1).

## 2 Analysis of the dataset

### 2.1 The analysis of the quantitative proteomics

**Fig. 2:**
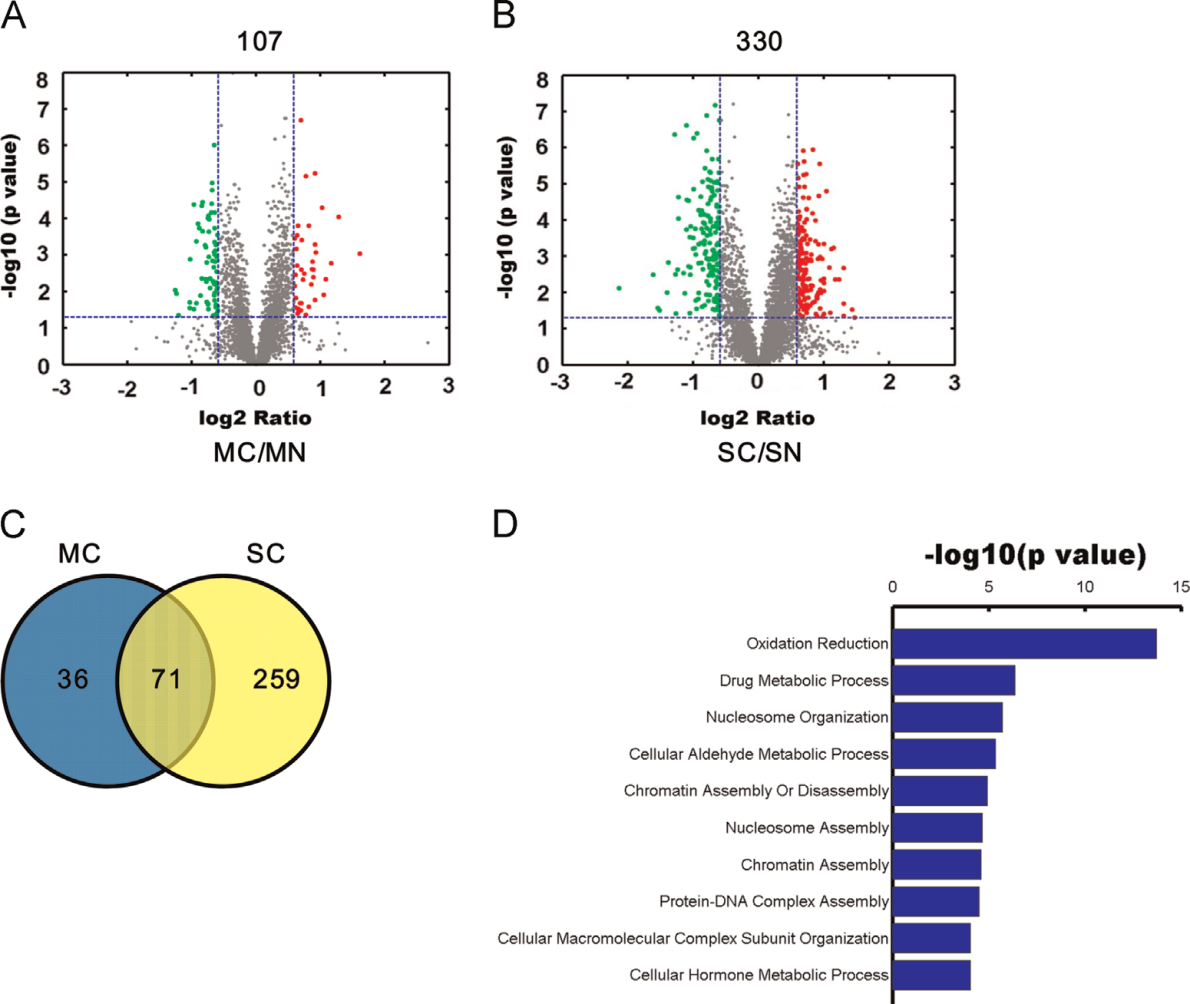
The iTRAQ ratio distribution and involved biological processes of the differentially expressed proteins in the HCC with single and multiple lesions. (A) Volcano plot represented the protein abundance changes in the HCC cancerous tissue with multiple lesions comparing to their adjacent noncancerous tissues. A total of 107 dysregulated proteins with fold change ≥1.5 and *p* values <0.05 were identified. (B) Volcano plot represented the protein abundance changes in the HCC cancerous tissues with a single lesion comparing to their adjacent noncancerous tissue. A total of 330 dysregulated proteins with fold change ≥1.5 and *p* values <0.05 were identified. (C) Venn diagrams showed the overlaps and number of differentially expressed proteins in the HCC with single and multiple lesions. (D) GO analysis of the involved biological processes of the common dysregulated proteins in both types of the HCC. All of the biological processes were ranked in term of enrichment of the differentially expressed proteins, and the top 10 are presented here.

In this dataset, 107 and 330 proteins were classified as differentially expressed in HCC tumor tissues with single and multiple lesions compared to surrounding noncancerous tissues (Fig. 2A, B). All of the differentially expressed proteins presented a mean expression fold change of ±1.5 (log_2_ 0.58) or even more with a *p* value less than 0.05 (paired *T*-test), meanwhile these proteins should have the same change trends in all six biological replicates. Among these differentially expressed proteins, 71 proteins altered their expression in both HCC types (Fig. 2C). GO annotation analysis showed that these proteins were the major participants in the oxidation reduction process and the cellular metabolic processes (Fig. 2D).

### 2.2 Bioinformatics analysis

**Fig. 3:**
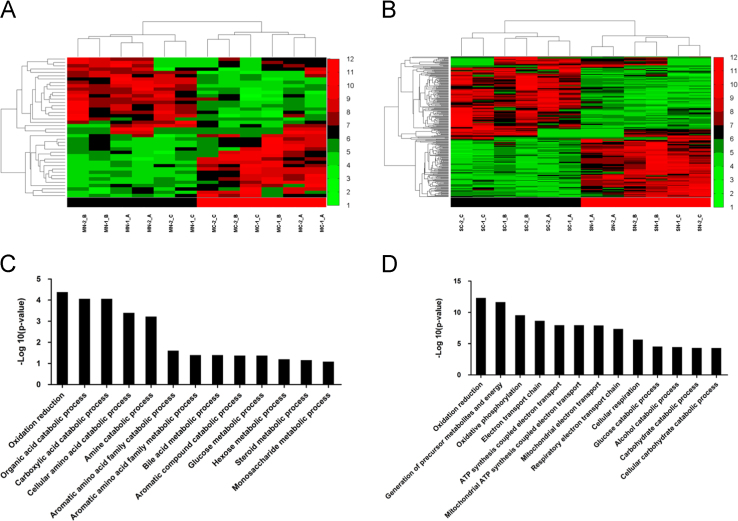
The hierarchical clustering and involved biological processes analysis of differentially expressed proteins in the primary HCC with single and multiple lesions. (A) Hierarchical clustering of the 107 dysregulated proteins in the HCC with multiple lesions (MC vs. MN). (B) Hierarchical clustering of the 330 dysregulated proteins in the HCC with a single lesion (SC vs. SN). (C, D) GO analysis of the dysregulated proteins involved biological processes in the HCC with multiple lesions (C) and in the HCC with a single lesion (D).

**Fig. 4:**
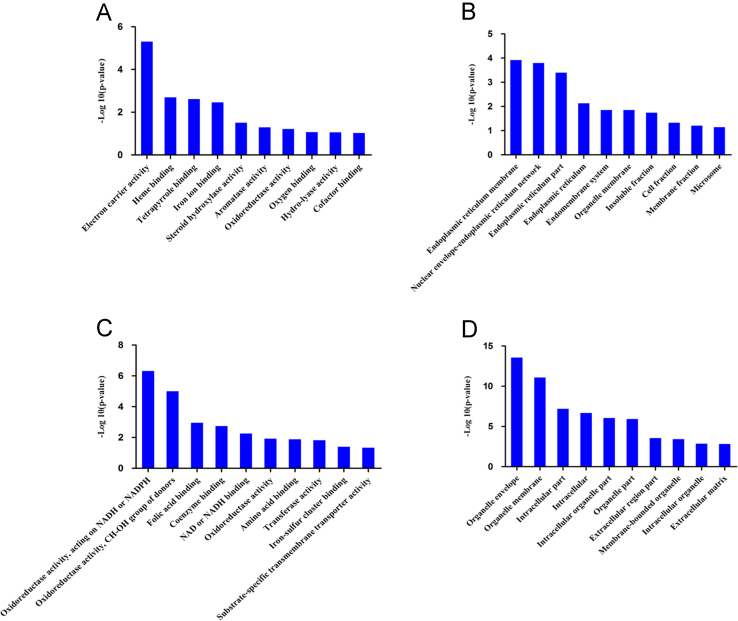
Gene ontology (GO) function analysis of differentially expressed proteins which is only dysregulated in HCC with a single lesion or HCC with multiple lesions. (A) GO analysis of the molecular function of the proteins which is only differentially expressed in HCC with multiple lesions. (B) GO analysis of the molecular function of the proteins which is only differentially expressed in HCC with a single lesion. (C) GO analysis of the cell component of the proteins which is only differentially expressed in HCC with multiple lesions. (D) GO analysis of the cell component of the proteins which is only differentially expressed in HCC with a single lesion. All of biological processes were ranked in term of the enrichment of the differentially expressed proteins, and the top 10 are presented.

The Gene Ontology (GO) annotation and pathway enrichment analysis of all the identified proteins and differentially expressed proteins were implemented using the online tool DAVID (http://david.abcc.ncifcrf.gov/). The quantitative iTRAQ ratios of 36 proteins, which dysregulated in MC group comparing to MN group, but these proteins were not dysregulated in primary HCC with a single lesion, were plotted on a heatmap (Fig. 3A). The names of the dysregulated proteins are listed in Table 2. We further analyzed these protein involved biological process by GO analysis (Fig. 3C). Meanwhile, 142 up-regulated proteins and 117 down-regulated proteins were specifically appeared in HCC with a single lesion group, but not in HCC with multiple lesions group; and the up and down regulated proteins also form clearly distinct clusters in the heatmap (Fig. 3B). The list of protein names is also displayed in Table 3. We further analyzed these protein involved biological process by GO analysis (Fig. 3D). Gene ontology (GO) analysis of the molecular function and cell component of differentially expressed proteins which is only dysregulated in HCC with a single lesion or HCC with multiple lesions are also displayed in Fig. 4.

**Fig. 5:**
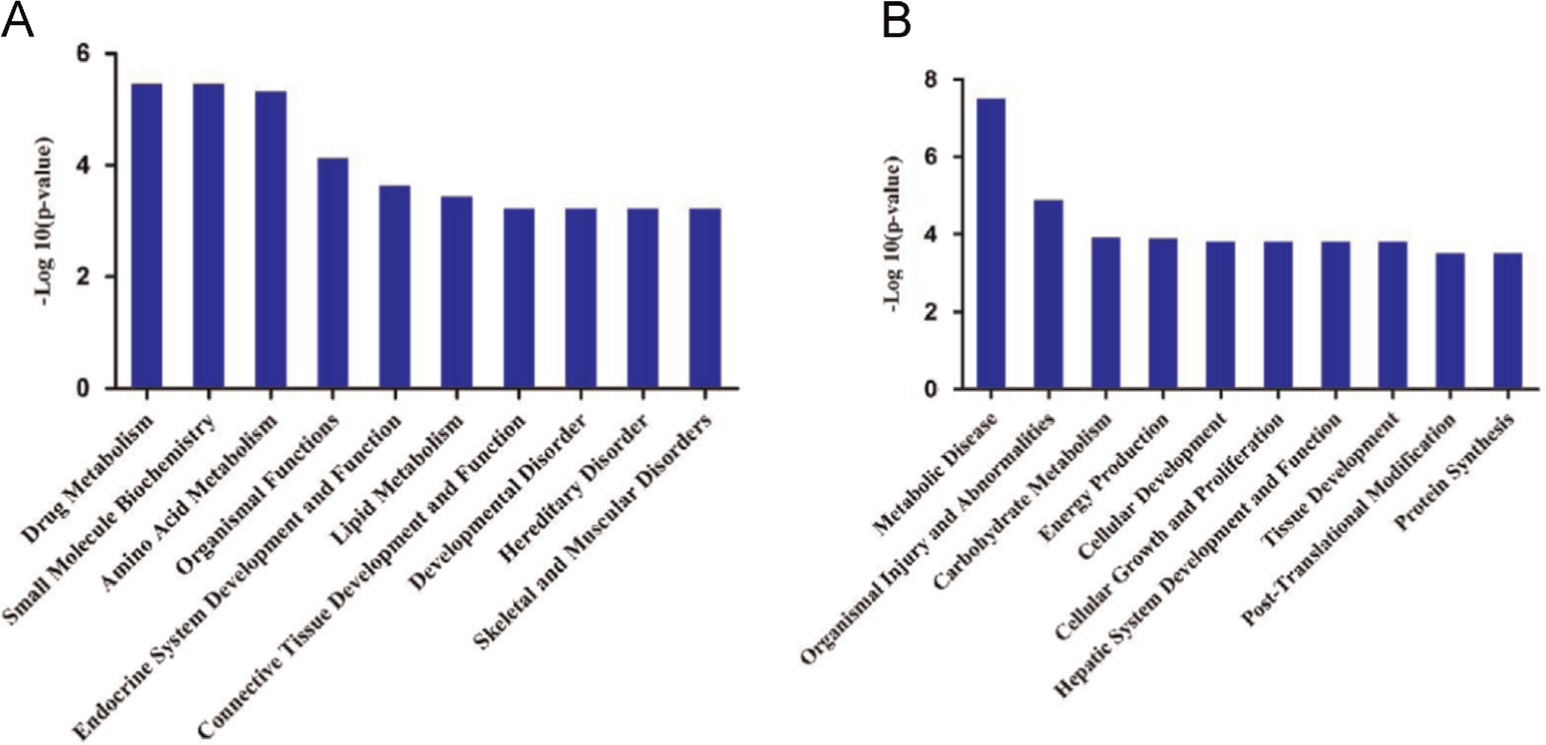
The key functions of the differentially expressed proteins involved in the HCC with single and multiple lesions according to IPA analysis. (A) Enriched Functions of the differentially expressed proteins that is only dysregulated in HCC with multiple lesions. (B) Enriched Functions of the differentially expressed proteins that is only dysregulated in HCC with a single lesion. All of pathways were ranked in term of the enrichment of the differentially expressed proteins, and the top 10 were presented.

The biological functions and signaling pathway annotations of the differentially expressed proteins were analyzed by Ingenuity Pathways Analysis (IPA) software (version 7.5), which is based on the Ingenuity Pathways database. The key functions of the differentially expressed proteins involved in the HCC with single and multiple lesions according to IPA analysis are also displayed in Fig. 5. The GO annotations, involved signaling pathways and networks were ranked in term of the enrichment of the differentially expressed proteins.

## Figures and Tables

**Table 1:** Basic information and characteristics of the HCC patients with a single or multiple observed lesions, who were enrolled in this dataset.

	HCC with multiple lesions	HCC with a single lesion
Gender		
Male	30	30
Female	0	0
		
Age (years)		
≤55	17	17
>55	13	13
		
AFP (ng/ml)		
≤400	12	20
>400	18	10
		
Tumor size (cm)		
≤5	9	6
5–10	21	24
		
Progression of cirrhosis		
None	3	5
Mild	13	12
Moderate	13	12
Severe	1	1
		
Tumor boundaries		
Distinct	18	22
Indistinct	12	8
		
Differentiation degree		
I–II	8	4
II–III	17	22
III–IV	5	4
		
Vascular tumor thrombosis		
No	26	25
Yes	4	5
		
Tumor encapsulation		
No	2	6
Incomplete	14	10
Complete	14	14

**Table 2:** List of the differentially expressed proteins which is only dysregulated in HCC with multiple lesions.

Differentially expressed proteins	Gene	Fold change	Fold change
MC/MN	SC/SN
UTP-glucose-1-phosphate uridylyltransferase	UGP2	0.62	0.68
Bile acyl-CoA synthetase	SLC27A5	0.58	0.67
Cytochrome P450 2A6	CYP2A6	0.6	0.73
Glycine dehydrogenase (decarboxylating), mitochondrial	GLDC	0.65	0.72
17-Beta-hydroxysteroid dehydrogenase 13	HSD17B13	0.56	0.71
Glycogen [starch] synthase, liver	GYS2	0.65	0.7
Sequestome-1	SQSTM1	1.77	1.59
4-Hydroxyphenylpyruvate dioxygenase	HPD	0.63	0.76
Kynurenine 3-monooxygenase	KMO	0.56	0.76
Beta-enolase	ENO3	0.64	0.71
Urocanate hydratase	UROC1	0.66	0.76
Keratin, type I cytkeletal 20	KRT20	1.53	1.97
Synembryn-A	RIC8A	1.56	1.4
Cadherin-related family member 2	CDHR2	0.64	0.78
Cytochrome P450 2B6	CYP2B6	0.64	0.69
NAD(P)H dehydrogenase [quinone] 1	NQO1	1.89	1.52
Anterior gradient protein 2 homolog	AGR2	1.71	1.14
Peripherin	PRPH	0.64	0.7
Fuce mutarotase	FUOM	0.6	0.61
Coiled-coil domain-containing protein 57	CCDC57	1.54	1.4
Gangliide-induced differentiation-associated protein 1	GDAP1	1.51	1.44
Histone H1.1	HIST1H1A	1.62	1.12
Choline transporter-like protein 2	SLC44A2	0.65	0.63
RAS protein activator like-3	RASAL3	0.63	0.79
Non-histone chromomal protein HMG-17	HMGN2	3.06	1.65
FH1/FH2 domain-containing protein 1	FHOD1	1.66	1.44
Copine-6	CPNE6	0.58	0.78
Myeloblastin	PRTN3	1.51	1.94
24-Hydroxycholesterol 7-alpha-hydroxylase	CYP39A1	0.61	0.71
Sodium/hydrogen exchanger 10	SLC9C1	0.63	0.68
Steroid 17-alpha-hydroxylase/17,20 lyase	CYP17A1	1.57	2.15
HLA class I histocompatibility antigen, alpha chain G	HLA-G	0.66	1.08
MICAL C-terminal-like protein	MICALCL	0.49	0.4
Nucleolysin TIA-1 isoform p40	TIA1	0.66	0.91
Immunoglobulin-binding protein 1	IGBP1	1.57	1.63
Protein FAM171A1	FAM171A1	0.59	0.63

**Table 3:** List of the differentially expressed proteins which is only dysregulated in HCC with a single lesion.

Differentially expressed proteins	Gene	Fold change	Fold change
MC/MN	SC/SN
Keratin, type II cytkeletal 8	KRT8	0.73	0.62
Keratin, type I cytkeletal 18	KRT18	0.72	0.6
Tenascin-X	TNXB	0.72	0.65
C-1-tetrahydrofolate synthase, cytoplasmic	MTHFD1	0.72	0.56
Trifunctional enzyme subunit beta, mitochondrial	HADHB	0.91	0.6
Acetyl-CoA acetyltransferase, mitochondrial	ACAT1	0.78	0.61
Long-chain-fatty-acid–CoA ligase 1	ACSL1	0.72	0.65
Haptoglobin	HP	0.68	0.5
3-Ketoacyl-CoA thiolase, mitochondrial	ACAA2	0.81	0.5
Non-specific lipid-transfer protein	SCP2	0.91	0.65
Lumican	LUM	0.86	0.63
Fatty acid-binding protein, liver	FABP1	0.68	0.6
d-Beta-hydroxybutyrate dehydrogenase, mitochondrial	BDH1	0.69	0.65
Betaine–homocysteine S-methyltransferase 1	BHMT	0.67	0.58
Putative hexokinase HKDC1	HKDC1	1.28	1.54
l-Lactate dehydrogenase A chain	LDHA	1.24	1.6
Pyruvate kinase PKM	PKM	1.25	1.59
X-ray repair crs-complementing protein 6	XRCC6	1.36	1.63
Enoyl-CoA hydratase, mitochondrial	ECHS1	0.86	0.55
Delta(3,5)-Delta(2,4)-dienoyl-CoA isomerase, mitochondrial	ECH1	0.87	0.65
ATP synthase subunit d, mitochondrial	ATP5H	0.78	0.56
Laminin subunit beta-1	LAMB1	1.42	1.53
S-adenylmethionine synthase isoform type-1	MAT1A	0.74	0.66
X-ray repair crs-complementing protein 5	XRCC5	1.49	1.77
Electron transfer flavoprotein subunit alpha, mitochondrial	ETFA	0.83	0.65
ATP-citrate synthase	ACLY	1.16	1.7
Myeloperoxidase	MPO	1.37	1.58
Glucose-6-phosphate isomerase	GPI	1.14	1.83
Villin-1	VIL1	1.36	1.75
Short/branched chain specific acyl-CoA dehydrogenase, mitochondrial	ACADSB	0.86	0.61
Endoplasmic reticulum resident protein 29	ERP29	0.77	0.59
Superoxide dismutase [Cu–Zn]	SOD1	0.83	0.64
C4b-binding protein alpha chain	C4BPA	1.24	1.62
DnaJ homolog subfamily B member 9	DNAJB9	0.81	0.45
3-Ketoacyl-CoA thiolase, peroxisomal	ACAA1	0.8	0.63
Decorin	DCN	0.8	0.64
Transketolase	TKT	1.42	1.66
Ferritin light chain	FTL	0.73	0.62
Elongation factor 1-gamma	EEF1G	1.18	1.55
Cytochrome b-c1 complex subunit 7	UQCRB	0.79	0.53
Transferrin receptor protein 1	TFRC	1.49	1.89
Glycerol-3-phosphate dehydrogenase [NAD(+)], cytoplasmic	GPD1	0.77	0.64
Peroxiredoxin-4	PRDX4	0.7	0.64
Mimecan	OGN	0.77	0.62
Cytochrome c oxidase subunit 6B1	COX6B1	0.79	0.58
NADH dehydrogenase [ubiquinone] flavoprotein 2, mitochondrial	NDUFV2	0.82	0.55
Phenylalanine-4-hydroxylase	PAH	0.68	0.66
6-Phosphogluconate dehydrogenase, decarboxylating	PGD	1.22	1.73
ATP synthase subunit O, mitochondrial	ATP5O	0.93	0.66
Cytochrome b-c1 complex subunit Rieske, mitochondrial	UQCRFS1	0.85	0.65
Cytochrome c oxidase subunit 5B, mitochondrial	COX5B	0.79	0.55
Dehydrogenase/reductase SDR family member 4	DHRS4	0.94	0.6
Gamma-glutamyltransferase 5	GGT5	0.67	0.6
Sulfotransferase 1A1	SULT1A1	0.81	0.58
Carboxypeptidase D	CPD	1.44	1.61
Spliceome RNA helicase DDX39B	DDX39B	1.18	1.53
Core histone macro-H2A.1	H2AFY	1.48	1.51
Polymerase I and transcript release factor	PTRF	0.83	0.62
Apolipoprotein D	APOD	0.84	0.66
ATP synthase-coupling factor 6, mitochondrial	ATP5J	0.78	0.56
Glucose-6-phosphate 1-dehydrogenase	G6PD	1.43	1.61
2-Oxoisovalerate dehydrogenase subunit alpha, mitochondrial	BCKDHA	0.83	0.63
NADH dehydrogenase [ubiquinone] 1 beta subcomplex subunit 10	NDUFB10	0.83	0.58
Glycine N-acyltransferase	GLYAT	0.73	0.6
Cytochrome c oxidase subunit 5A, mitochondrial	COX5A	0.82	0.59
DNA replication licensing factor MCM3	MCM3	1.5	1.62
Ribonuclease UK114	HRSP12	0.82	0.65
Phenazine biosynthesis-like domain-containing protein	PBLD	0.72	0.58
Asparagine–tRNA ligase, cytoplasmic	NARS	1.43	1.67
Lamin-B receptor	LBR	1.36	1.68
Polypeptide N-acetylgalactaminyltransferase 2	GALNT2	1.22	1.58
Paralemmin-3	PALM3	0.71	0.63
EGF-containing fibulin-like extracellular matrix protein 1	EFEMP1	1.37	1.51
Heme-binding protein 1	HEBP1	0.83	0.54
Apolipoprotein C-III	APOC3	0.92	0.66
Phosphoglucomutase-2	PGM2	1.16	1.85
Complement factor H-related protein 5	CFHR5	0.89	0.63
l-Lactate dehydrogenase B chain	LDHB	1.2	1.62
Serine–tRNA ligase, cytoplasmic	SARS	1.09	1.55
ATP synthase subunit e, mitochondrial	ATP5I	0.79	0.56
Creatine kinase B-type	CKB	0.95	1.92
NADH dehydrogenase [ubiquinone] 1 alpha subcomplex subunit 5	NDUFA5	0.74	0.53
NADH dehydrogenase [ubiquinone] iron–sulfur protein 8, mitochondrial	NDUFS8	0.89	0.66
Desmin	DES	0.71	0.51
DNA replication licensing factor MCM6	MCM6	1.34	1.54
Serum deprivation-response protein	SDPR	0.79	0.55
Acyl-coenzyme A synthetase ACSM3, mitochondrial	ACSM3	0.75	0.6
Clathrin light chain B	CLTB	0.88	0.65
Probable d-lactate dehydrogenase, mitochondrial	LDHD	0.75	0.63
Beta-2-microglobulin	B2M	0.87	0.63
Four and a half LIM domains protein 1	FHL1	0.86	0.66
NADH dehydrogenase [ubiquinone] 1 alpha subcomplex subunit 2	NDUFA2	0.85	0.58
Perilipin-2	PLIN2	1.23	1.65
NADH dehydrogenase [ubiquinone] 1 alpha subcomplex subunit 8	NDUFA8	0.72	0.47
Tubulointerstitial nephritis antigen-like	TINAGL1	0.81	0.61
Farnesyl pyrophosphate synthase	FDPS	1.37	1.6
Minor histocompatibility antigen H13	HM13	1.3	1.68
Glutathione peroxidase 1	GPX1	0.81	0.64
DNA-(apurinic or apyrimidinic site) lyase	AX1	1.34	1.67
Procollagen-lysine, 2-oxoglutarate 5-dioxygenase 3	PLOD3	1.15	1.58
Angiotensinogen	AGT	1.14	1.55
Transmembrane protein 2	TMEM2	1.1	1.51
Alpha/beta hydrolase domain-containing protein 14B	ABHD14B	0.86	0.66
EF-hand domain-containing protein D1	EFHD1	0.81	0.65
Protein mago nashi homolog 2	MAGOHB	1.3	1.73
3-Hydroxyanthranilate 3,4-dioxygenase	HAAO	0.79	0.66
Cofilin-2	CFL2	0.78	0.64
NADH dehydrogenase [ubiquinone] iron–sulfur protein 6, mitochondrial	NDUFS6	0.82	0.47
NADH dehydrogenase [ubiquinone] 1 alpha subcomplex subunit 12	NDUFA12	0.8	0.62
Alpha-fetoprotein	AFP	1.02	2.13
Proliferating cell nuclear antigen	PCNA	1.43	1.62
Transmembrane 9 superfamily member 4	TM9SF4	1.35	1.54
NADH dehydrogenase [ubiquinone] iron–sulfur protein 5	NDUFS5	0.82	0.56
Nuclear cap-binding protein subunit 1	NCBP1	1.34	1.55
Dermatopontin	DPT	0.74	0.62
Glycine N-methyltransferase	GNMT	0.8	0.65
Ataxin-10	ATXN10	1.38	1.57
UPF0553 protein C9orf64	C9orf64	1.41	1.64
BRO1 domain-containing protein BROX	BROX	1.38	1.51
NADH dehydrogenase [ubiquinone] iron–sulfur protein 4, mitochondrial	NDUFS4	0.78	0.5
l-Serine dehydratase/l-threonine deaminase	SDS	1.01	0.65
Protein transport protein Sec23B	SEC23B	1.39	1.65
Mitochondrial import inner membrane translocase subunit Tim8 A	TIMM8A	0.82	0.56
Nicastrin	NCSTN	1.29	1.53
Cytochrome P450 3A7	CYP3A7	1.38	1.94
40S ribomal protein S15	RPS15	1.08	0.55
Integrin alpha-IIb	ITGA2B	1.1	0.65
Acyl-CoA:lysophosphatidylglycerol acyltransferase 1	LPGAT1	1.14	1.51
Apolipoprotein L1	APOL1	1.36	1.61
Peptidyl-prolyl cis–trans isomerase FKBP2	FKBP2	0.9	0.66
Complement factor H-related protein 1	CFHR1	0.7	0.63
Plasma serine protease inhibitor	SERPINA5	1.08	1.52
Mitochondrial import inner membrane translocase subunit Tim13	TIMM13	0.81	0.48
Tropomodulin-1	TMOD1	0.83	0.64
Myin regulatory light polypeptide 9	MYL9	0.82	0.62
ER lumen protein retaining receptor 1	KDELR1	1.1	1.58
NAD-dependent malic enzyme, mitochondrial	ME2	1.33	1.6
Ceramide synthase 2	CERS2	1.47	1.51
Monocarboxylate transporter 4	SLC16A3	1.44	1.66
Glutaredoxin-1	GLRX	0.82	0.66
Collagen alpha-6(VI) chain	COL6A6	0.75	0.63
Group XIIB secretory phospholipase A2-like protein	PLA2G12B	0.79	0.59
Latent-transforming growth factor beta-binding protein 2	LTBP2	1.33	1.53
Myin-7	MYH7	1.14	2.46
15 kDa selenoprotein	15-Sep	0.69	0.58
Metalloproteinase inhibitor 1	TIMP1	1.33	1.52
Protein RCC2	RCC2	1.37	1.65
NADH dehydrogenase [ubiquinone] 1 alpha subcomplex subunit 7	NDUFA7	0.86	0.57
Calmegin	CLGN	1.41	1.75
Apolipoprotein(a)	LPA	0.74	0.64
Elongation factor 1-alpha 2	EEF1A2	1.13	1.74
Cytochrome c oxidase protein 20 homolog	COX20	1.38	1.63
Translin	TSN	1.2	1.57
Folate receptor beta	FOLR2	0.72	0.65
Secretory carrier-associated membrane protein 3	SCAMP3	1.38	1.53
Chitinase-3-like protein 1	CHI3L1	1.11	1.86
Mitochondrial intermembrane space import and assembly protein 40	CHCHD4	0.9	0.57
Fibrocystin-L	PKHD1L1	0.71	0.55
Girdin	CCDC88A	0.82	0.38
Flap endonuclease 1	FEN1	1.3	1.74
Solute carrier family 43 member 3	SLC43A3	1.29	1.65
Complement factor H-related protein 3	CFHR3	0.84	0.59
Cleavage stimulation factor subunit 3	CSTF3	1.39	1.88
Protein kinase C delta-binding protein	PRKCDBP	0.76	0.54
Transmembrane protein 176B	TMEM176B	1.32	1.62
60 kDa lysophospholipase	ASPG	0.7	0.62
Spermidine synthase	SRM	1.2	1.51
B-cell receptor-associated protein 29	BCAP29	1.11	1.56
Retinol dehydrogenase 10	RDH10	1.36	1.58
PDZ and LIM domain protein 2	PDLIM2	0.8	0.6
Sodium/potassium-transporting ATPase subunit beta-3	ATP1B3	1.46	1.56
Uncharacterized protein C19orf52	C19orf52	1.36	1.53
Transmembrane protein 70, mitochondrial	TMEM70	1.24	1.51
Insulin-like growth factor 2 mRNA-binding protein 2	IGF2BP2	1.41	1.77
C-reactive protein	CRP	1.2	1.83
Importin subunit alpha-1	KPNA2	1.33	2.77
COX assembly mitochondrial protein 2 homolog	CMC2	0.82	0.61
Retinoic acid receptor responder protein 2	RARRES2	1.38	1.54
Oncoprotein-induced transcript 3 protein	OIT3	0.71	0.59
Ficolin-1	FCN1	0.9	0.65
StAR-related lipid transfer protein 5	STARD5	0.76	0.56
Transmembrane protein 14C	TMEM14C	1.33	1.64
P2X purinoceptor 4	P2RX4	1.39	1.58
Bifunctional lysine-specific demethylase and histidyl-hydroxylase MINA	MINA	1.28	1.63
Myeloid leukemia factor 2	MLF2	0.81	0.58
C4b-binding protein beta chain	C4BPB	1.16	1.65
Astrocytic phosphoprotein PEA-15	A15	1.07	1.53
Pituitary tumor-transforming gene 1 protein-interacting protein	PTTG1IP	1.36	1.68
Unconventional myin-XIX	MYO19	1.4	1.55
Ras-related protein Rab-3D	RAB3D	1.49	1.61
F-box only protein 22	FBXO22	1.15	1.61
UPF0364 protein C6orf211	C6orf211	1.33	1.59
PRA1 family protein 2	PRAF2	1.28	1.53
Serine incorporator 1	SERINC1	1.41	1.53
Spermatogenesis-defective protein 39 homolog	VIPAS39	1.33	1.53
Ryanodine receptor 1	RYR1	0.91	1.52
AP-1 complex subunit gamma-like 2	AP1G2	1.4	1.65
Hexokinase-2	HK2	1.37	1.97
Uncharacterized protein C2orf42	C2orf42	1.19	1.53
Phospholipid transfer protein	PLTP	1.05	2.05
PC4 and SFRS1-interacting protein	PSIP1	1.3	1.53
Rho guanine nucleotide exchange factor 18	ARHGEF18	1.48	1.56
Acylphosphatase-2	ACYP2	0.82	0.6
Claudin-1	CLDN1	1.05	1.68
Neutral amino acid transporter B(0)	SLC1A5	1.2	2
CB1 cannabinoid receptor-interacting protein 1	CNRIP1	0.91	0.66
Cytolic Fe–S cluster assembly factor NUBP2	NUBP2	0.84	0.6
Sortilin	SORT1	1.12	1.51
UPF0729 protein C18orf32	C18orf32	1.15	1.77
Protein YIPF4	YIPF4	1.33	1.6
Cell growth regulator with EF hand domain protein 1	CGREF1	1.11	1.54
Presenilins-associated rhomboid-like protein, mitochondrial	PARL	1.3	1.59
Bactericidal permeability-increasing protein	BPI	1.23	1.66
Protein S100-A1	S100A1	1.27	1.63
Ammonium transporter Rh type A	RHAG	2.34	1.83
Pleckstrin homology domain-containing family G member 3	PLEKHG3	1.37	1.52
Putative methyltransferase NSUN5	NSUN5	0.71	0.59
Secretory carrier-associated membrane protein 4	SCAMP4	1.32	1.61
Cochlin	COCH	1.31	2.12
tRNA (guanine(10)-N2)-methyltransferase homolog	TRMT11	1.33	2
Protein YIPF3	YIPF3	1.27	1.54
Synaptogyrin-1	SYNGR1	1.34	1.73
Ubiquitin carboxyl-terminal hydrolase isozyme L1	UCHL1	1.26	1.57
MAP kinase-activated protein kinase 2	MAPKAPK2	1.41	1.52
Proteoglycan 3	PRG3	0.69	0.65
Bcl-2 homologous antagonist/killer	BAK1	1.3	1.7
TNF receptor-associated factor 6	TRAF6	1.03	0.66
Ethanolamine-phosphate phospho-lyase	ETNPPL	0.66	0.46
Proto-oncogene tyrine-protein kinase Src	SRC	1.29	1.62
Folate transporter 1	SLC19A1	1.3	1.81
Platelet factor 4	PF4	0.76	0.54
Chloride intracellular channel protein 5	CLIC5	1	1.52
Negative elongation factor E	NELFE	1.32	1.64
RNA polymerase II-associated protein 1	RPAP1	1.26	1.65
Zinc transporter SLC39A7	SLC39A7	1.67	2
Ankyrin repeat domain-containing protein 24	ANKRD24	0.8	1.65
Centromal protein of 85 kDa-like	CEP85L	0.26	0.65
Caspase-3	CASP3	1.22	1.68
Peroxisomal leader peptide-processing protease	TYSND1	1.3	1.6
tRNA (guanine(37)-N1)-methyltransferase	TRMT5	1.26	1.86
Mitochondrial inner membrane organizing system protein 1	MINOS1	1.31	1.52
Coiled-coil domain-containing protein 153	CCDC153	1.36	2.47
Conserved oligomeric Golgi complex subunit 8	COG8	1.23	1.56
Vesicle transport protein SFT2B	SFT2D2	1.13	1.51
F-box only protein 10	FBXO10	0.71	0.63
Muskelin	MKLN1	1.09	1.57
Tuftelin-interacting protein 11	TFIP11	1.27	1.52
Sulfotransferase 1C2	SULT1C2	1.15	1.62
Zinc transporter ZIP1	SLC39A1	1.45	1.91
Proton-coupled folate transporter	SLC46A1	1.19	1.52
Putative heat shock protein HSP 90-beta 2	HSP90AB2P	1.29	2.25
Asparagine synthetase [glutamine-hydrolyzing]	ASNS	1.21	2.7
Ubiquitin carboxyl-terminal hydrolase 38	USP38	0.64	0.52
Glycogen synthase kinase-3 alpha	GSK3A	1.34	1.71
Coiled-coil domain-containing protein 69	CCDC69	0.8	0.66
Retinoid-binding protein 7	RBP7	0.95	1.51
Signal-transducing adaptor protein 2	STAP2	1.21	1.59
Soluble calcium-activated nucleotidase 1	CANT1	1.5	2
Threonine synthase-like 2	THNSL2	0.65	0.48
